# Using stable isotopes as tracer to investigate hydrological condition and estimate water residence time in a plain region, Chengdu, China

**DOI:** 10.1038/s41598-021-82349-3

**Published:** 2021-02-02

**Authors:** Jing Zhou, Guodong Liu, Yuchuan Meng, ChengCheng Xia, Ke Chen, Yu Chen

**Affiliations:** 1 State Key Laboratory of Hydraulics and Mountain River Engineering, Sichuan University, Chengdu, 610065 China; 2 College of Water Resources and Hydropower, Sichuan University, Chengdu, 610065 China

**Keywords:** Hydrology, Environmental impact

## Abstract

The oxygen and hydrogen isotopic compositions (δ^18^O and δ^2^H) were measured on river water and precipitation collected from four sub-catchments within the upper Tuojiang River catchment. δ^18^O values of river water and precipitation exhibit significant seasonal variations. These seasonal variations are used for estimating the mean residence time (MRT) for four sub-catchments by an exponential model, ranging from 346 to 493 days. The correlation between catchment MRT and mean slope of the catchment (r^2^ = 0.29) is weak, while the correlations between catchment MRT, catchment area (r^2^ = 0.79) and topographic index (r^2^ = 0.98) are strong. These results indicate that topography and catchment area, both control the catchment MRT and the topographic index may be a reliable parameter for estimating the catchment MRT. Moreover, the relationship between land use types and MRT was investigated. The results show that paddy fields (r^2^ = 0.95) compared to the other land use types may have a greater impact on the MRT of the irrigation-dominated catchment. This study provides a preliminary exploration of the factors affecting MRT in the plain region and a basis for simulating MRT in the future.

## Introduction

The catchment hydrological process is complex and it is difficult to observe how rivers respond to precipitation events. Over the past three decades, hydrologists are starving to figure out how to improve our understanding of the process and quantitative knowledge at scales where water resource decision was obtained^1^. As advances in measurement techniques are achieved, isotope technology provides important insight in better understanding the hydrological process^2–4^. The residence time refers to the time water travels through the subsurface of the catchment after its input as precipitation^5^. MRT is an important descriptor of catchment hydrology to indicate storage, flow path and source of water in an integrated measurement^6^. Damping of tritium (^3^H/^1^H), variations of stable oxygen (^18^O/^16^O) and hydrogen (^2^H/^1^H) isotopic signature in both precipitation and river water have been proved useful for estimating the MRT of catchment^7–10^. The distribution of water residence time in catchments is not only a reflection of flow pathway and storage, but also an important indicator for water quality as biogeochemical reaction relates directly to time^11^. Longer residence time means longer contact time with catchment. Therefore, by quantifying the MRT, important information about hydro-chemical systems, hydrological sensitivity to human activities and climatic changes could be revealed^12,13^. Knowledge of residence time would also help to investigate how a catchment stores and releases water as it is associated with the diversity of flow pathways in catchment^14,15^. Therefore, there have been numerous interests recently in estimating catchment residence time to describe catchment hydrology^16–18^. Besides, many influence factors relate to MRT have been analysed in many studies. For example, Farrick and Branfireun^17^ have found that there is a strong negative relationship between MRT and catchment slope, while a positive relationship between mean flow path length and MRT is observed in a forested tropical catchment. Topographic characteristic like the mean topographic index, which is an attribute that describes the surface flow path, is strongly correlated with MRT^7^. As the existence of lake storage, MRT could vary from days to years^16^. Stewart and Mcdonnell^19^ have found the increasing tendency of MRT with soil depth, which indicates the soil depth may also be a factor related to MRT. To date, many studies are interested in estimating MRT for characterizing the catchment. Most of them focus on small catchments because of the difficulty that exists in field sampling and observation. Describing hydrological processes for large-scale catchments is important. Because it is necessary for developing a more conceptual model and be used to address questions at scale that impact land management and climate change^7^. Therefore, how to extend those results from a single small catchment to a large-catchment scale is a vital problem. The relationship between stream residence time and landscape feature provides an opportunity to resolve it. Rodgers et al.^1^, McGlynn et al.^8^ have proved that the residence time of water in the catchment is independent of the catchment scale. However, Farrick and Branfireun^17^ have found a strong negative relationship between the catchment area and MRT. Different results above may suggest that the topography or catchment area is not the only factor governing residence time distributions. In steep hillslope, the topography may mask the effect of the catchment area. Therefore, studies in the plain region with moderate terrain may help us to figure out how the catchment scale influences MRT. However, previous studies in plain referred to MRT focus on using MRT as a tool to investigate catchment hydrology rather than studying the relationship between MRT and catchment scale^20,21^. For example, Cao et al.^22^ estimated the MRT of groundwater in the Yang-Dai River plain to identify pollution status and groundwater cyclical pattern. Ben Ammar et al.^23^ carried out isotopic (^18^O, ^2^H and ^3^H) investigation to assesses groundwater renewability by estimate the residence time of groundwater in the plain of Wadi Guenniche. Ma and Yamanaka^24^ estimated time-variant MTTs for about ten years (2003–2012) in five mesoscale sub-catchments of the Fuji River catchment, central Japan and found long-term average MTT was principally controlled by the amount of groundwater storage, especially in the plain region.

In this paper, the four tributaries of the upper Tuojiang river in Chengdu plain was selected for its slight topography and relatively uniform climatic condition. We conducted a 1-year sampling and isotope measurement of the water from the four sub-catchments within the Tuojiang River catchment to analyze the periodicity of its isotope changes. Then, MRT values are estimated for the four sub-catchments, respectively. This paper aims to: (1) compare the MRT differences in the four sub-catchments of the upper Tuojiang River based on stable isotope data; (2) identify the correlation between the catchment parameters and MRT; and (3) determine the dominantly influencing factors of the MRT.

## Study area

**Figure 1:**
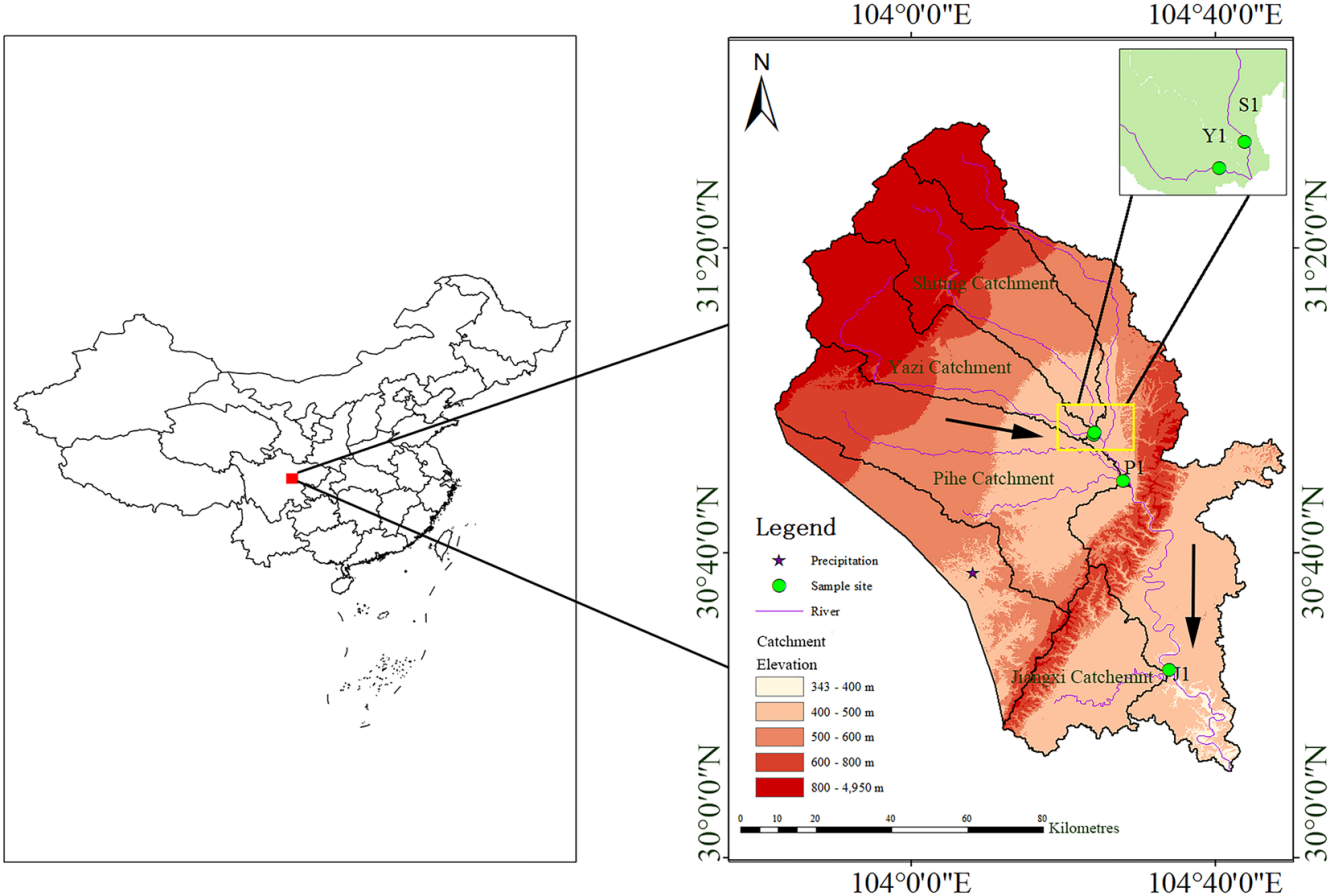
Map of the Tuojiang River catchment and its four sub-catchments, showing the location of the study area and the sampling site. S1, Y1, P1 and J1 are river water samples site and its elevations are 425 m for S1, 447 m for Y1, 440 m for P1 and 382 m for J1 respectively. The pentacle is the site for sampling precipitation (489 m). The source of the DEM based on is the open source data of the geospatial data cloud (http://www.gscloud.cn/sources/accessdata/305?pid=302) of which the spatial resolution is 90 m. This map was extracted and generated using ARCGIS (Version 10.2).

The Chengdu Plain is located in the west of the Sichuan Basin and surrounded by Longquan and Longmen mountains. As an alluvial plain in southwest China, it has an extensive area and its terrain is high in the west and low in the east with a low elevation. Tuojiang River is an upstream tributary of the Yangtze River and is an economically and ecologically important river passing through the hinterland of the Sichuan Basin (Fig. 1). It originates from Jiuding mountain in Mianzhu City and flows through the Chengdu Plain with a total length of 712 km and a drainage area of 32,900 km^2^^25^. The climate of Tuojiang River catchment is subtropical monsoon climate, with an annual average temperature of 17.1 °C, an annual average flow of 35.1 × 10^8^ m^3^ and average precipitation of 1050 mm. There are distinct dry and wet season with 65–75% of the total annual precipitation falls from June to September in the Tuojiang River catchment^26^. The Tuojiang River spans across several different terrains such as plain, hill and mountains and the plain are located along both sides of the Tuojiang River. This study focuses on four sub-catchments within Tuojiang River catchment (104°E–105.5°E, 29°N–31.5°N), named Shiting River catchment, Yazi River catchment, Pihe River catchment and Jiangxi River catchment respectively. Both Shiting River and Yazi River receive precipitation from 900 to 1500 mm annually, while the Pihe River and Jiangxi River receive only 870 mm. The four sub-catchments area range from 907.12 to 2168.55 km^2^ (Table 1). The Pihe River catchment, which is the largest catchment of these sub-catchments, is the lowest slope and paddy-dominated region (account for 63.96% of its total area) (Table 2). Pihe River originates from the Min River and flows into the Tuojiang River after merging with the Qingbai River. It connects the Min River system with the Tuojiang River system^27^. Slopes in the uppermost part of the Tuojiang River catchment (Shiting River catchment and Yazi River catchment) tend to be steep (13.44° to 14.03°) and slopes in the middle part of the Tuojiang River catchment (Pihe River catchment and Jiangxi River catchment) appear to be much gentler (2.21° to 3.39°). The Shiting River catchment and Yazi River catchment locate within the transition region between the Longmen mountain and the Chengdu plain, with the forest coverage range from 37.13 to 33.99%. The Jiangxi River is the first-grade tributary of the Tuojiang River. It originates from the Longquan Mountains and joins the Tuojiang River in Jianyang city. It is a typical seasonal river which is mainly recharged by rainfall and its water volume is quite small in the dry season.

**Table 1:** Topographic characteristics of the four sub-catchments within Tuojiang River catchment.

Catchment	Area (km^2^)	Mean slope (°)	Mean topographic index	Sand clay loam (%)
Yazi River	1329.24	14.03	8.26	46.90
Shiting River	1531.17	13.44	8.40	42.83
Pihe River	2168.55	2.21	10.58	78.17
Jiangxi River	907.12	6.39	8.05	22.17

**Table 2:** Statistics of the land use at four sub-catchments within Tuojiang River catchment.

Catchment	Resident and industrial area (%)	Forest land (%)	Cultivated land (%)	Paddy field (%)	Dry land (%)
Yazi River	5.21	37.13	47.47	38.44	9.03
Shiting River	6.41	33.99	49.28	41.34	7.94
Pihe River	17.96	9.42	70.86	63.96	9.68
Jiangxi River	1.65	16.52	79.07	32.30	46.77

Perudic Argosols (PA), Stagnic Anthrosols (SA), Dystric Purpli-Udic Cambosols (DPC), Typic Purpli-Udic Cambosols (TPC), Calcific Purpli-Udic Cambosols (CPC) and, Ochri-Aquic Cambosol (OC) are widely distributed on the study region. Tuojiang River flows through a densely populated region where various land types exist, including arid land, paddy field, orchard, forestland, grassland and shrub land^28^. The agricultural land is mainly distributed on the side of the mainstream of the Tuojiang river and the forestland is mainly distributed on the Mountain.

## Methods and materials

### Sampling and isotope analysis

A total of 113 precipitation samples were collected from May 2018 to April 2019 in bulk collectors in the test field of Sichuan University (104.08°E, 30.63°N) where the shortest distance from the Tuojiang River is 38 km. Precipitation samplers consist of plastic funnels that are attached to plastic bottles and a ball that is placed in the center of the funnel to minimize evaporation. Precipitation samples were immediately collected after every rainfall event to reduce the impact on evaporation to the minimum. The source of soil data comes from the Harmonized World Soil Database (HWSD) constructed by the Food and Agriculture Organization of the United Nations (FAO) and the International Institute for Applied Systems (IIASA) on 2009. The soil data resolution is 1 km^2^. The source of land use data comes from the Resource and Environment Science and Data Center of the Institute of Geographic Sciences and Resources Research, Chinese Academy of Sciences on 2015. The resolution of land use data is 1 km^2^.

A total of 116 river water samples were collected at the outlet section of four sub-catchments from May 2018 to April 2019. River water samples were hand-dipped along the river shore at a water depth below 20 cm where relatively clean and flowing water was presented. These water samples were stored in a 20 ml vial of the clean high-density polyethylene bottle with a tight screw cap and the water samples were quickly stored in the refrigerator. The river water samples were collected weekly during the wet season (May–October), while bi-weekly during the dry season (November–April). The number of samples per month may vary slightly due to unfavourable weather conditions and human factors. The isotopic composition (δ^18^O and δ^2^H) of the samples were determined at the Sichuan University laboratory, using a triple-liquid water isotope analyzer which is manufactured by Los Gatos Research (LGR). The LGR analyzer uses spectrometry to measure the stable isotope content of water and its measurement principle is the OFF-AXIS Integrated Cavity Output Spectrometer (OA-ICOS). With the aid of a fully automatic sampler, the analyzer continuously measures the sample according to a computer program and filters the sample impurities by using a 0.45 μm filter before measurement. Each sample was measured six times, the first two measurements were discarded due to their large error and only the average of the last four measurements was taken. Reference standard samples based on VSOMW-2 and SLAP-2 were measured at intervals of three water samples to diagnose whether an abnormality occurred during the measurement. The stable hydrogen and oxygen isotope values in the water sample are expressed by the thousandth deviation from the Vienna standard average seawater V-SMOW, which is defined as follows:

1 $$\updelta \left( {\permil} \right) = {{\left( {{{\text{R}}_{{\text{sample}}}} - {{\text{R}}_{{\text{standard}}}}} \right)} / {\left( {{{\text{R}}_{{\text{standard}}}}} \right)}} \times 100$$

where ${\text{R}}_{\text{sample}}$ and ${\text{R}}_{\text{standard}}$ represent the value of ^18^O/^16^O and ^2^H/^1^H in the samples and the standard samples respectively. The measurement error of the instrument is δ^2^H < 0.3‰, δ^18^O < 0.08‰.

### Theoretical methods

The isotope composition of river water response to the isotope composition of precipitation that falls on catchment need some time before reaching the river. The seasonal variability is reflected in stable isotope values of precipitation and is often inherited by river water. The river outflows composition at any time, ${\updelta}_{\text{out}}(t)$, consisting of past inputs lagged, ${\updelta}_{\text{in}}{(\text{t}-\uptau)}$, according to their residence time distribution, $\text{g}(\uptau)$^29,30^:

2 $${\updelta _{{\text{out}}}} = \int\limits_0^\infty  {{\text{g}}\left( \uptau  \right){\updelta _{{\text{in}}}}\left( {{\text{t}} - \uptau } \right){\text{d}}\uptau }$$

where $\uptau$ is the lag times between input and output tracer composition. Equation (2) is similar to the linear systems approach used in unit hydrograph models (Dooge, 1973). In this paper, only isotope tracer is considered, therefore the transfer function is represented by $\text{g} (\uptau)$. The approach mentioned above (Eq. (2)) is valid only in the circumstance of a steady system and a stable mean flow pattern^31^. The residence time distribution (ie. $\text{g}(\uptau)$) characterizes the fractional weighting of how isotope tracer exits the catchment. The MRT of tracer is equal to the MRT of water because we use the stable isotopes like ^2^H and ^18^O which are conservative as tracer^7^. Residence time distribution in catchment is selected by the exact nature of its flow path distribution and flow system. Three main model types, including dispersion model (DM), the exponential model (EM) and the exponential-piston flow model (EPM), have been used frequently in catchment systems^8,19,32–34^. The exponential model that has been proved valid in many catchments^35,36^ is employed in the study. The exponential model is defined by following the $\text{g} (\uptau)$ function^10^:

3 $${\text{g}}\left(\uptau\right)= {\text{T}}^{-1}{\text{exp}}\left(\uptau/{\text{T}}\right)$$

where T is the residence time.

In the middle latitude region, the stable isotopic compositions of precipitation have a strong seasonal change, especially in the monsoon climate zone like the southwest of China. Seasonal trends in ${\updelta}^{18}{\text{O}}$ value are modelled through periodic regression analysis to fit seasonal sine wave curves to annual ${\updelta}^{18}{\text{O}}$ value variations of the precipitation and river water^1,18,32^ defined as:

4 $${\updelta}^{18}\text{O=X+A}\left[{\text{cos}}\left(\text{ct}-\uptheta\right)\right]$$

where δ^18^O refers to the simulated δ^18^O, X refers to the annual average of the measured δ^18^O value, A for the annual amplitude of the measured value δ^18^O, c for the annual fluctuation of the radial frequency (0.017214 rad d^−1^), and t is the number of days between the sampling time and the starting time (2018/5/6), θ refers to the phase lag or time at which δ^18^O peaks.

The MRT is estimated via the commonly used exponential model in which precipitation inputs are assumed to mix rapidly with resident water, by the following equation:

5 $$\text{MRT} = {\text{c}}^{-1}{\text{[}{\left({\text{A}}_{\text{Z2}}/{\text{A}}_{\text{Z1}}\right)}^{-2}{-1]}}^{0.5}$$

In this equation, A_Z1_ is the amplitude of rainfall, A_Z2_ is the amplitude of the river water, and c is the radial frequency of the annual amplitude of Eq. (4).

## Results and discussion

### Stable isotope variation of rivers water

**Figure 2:**
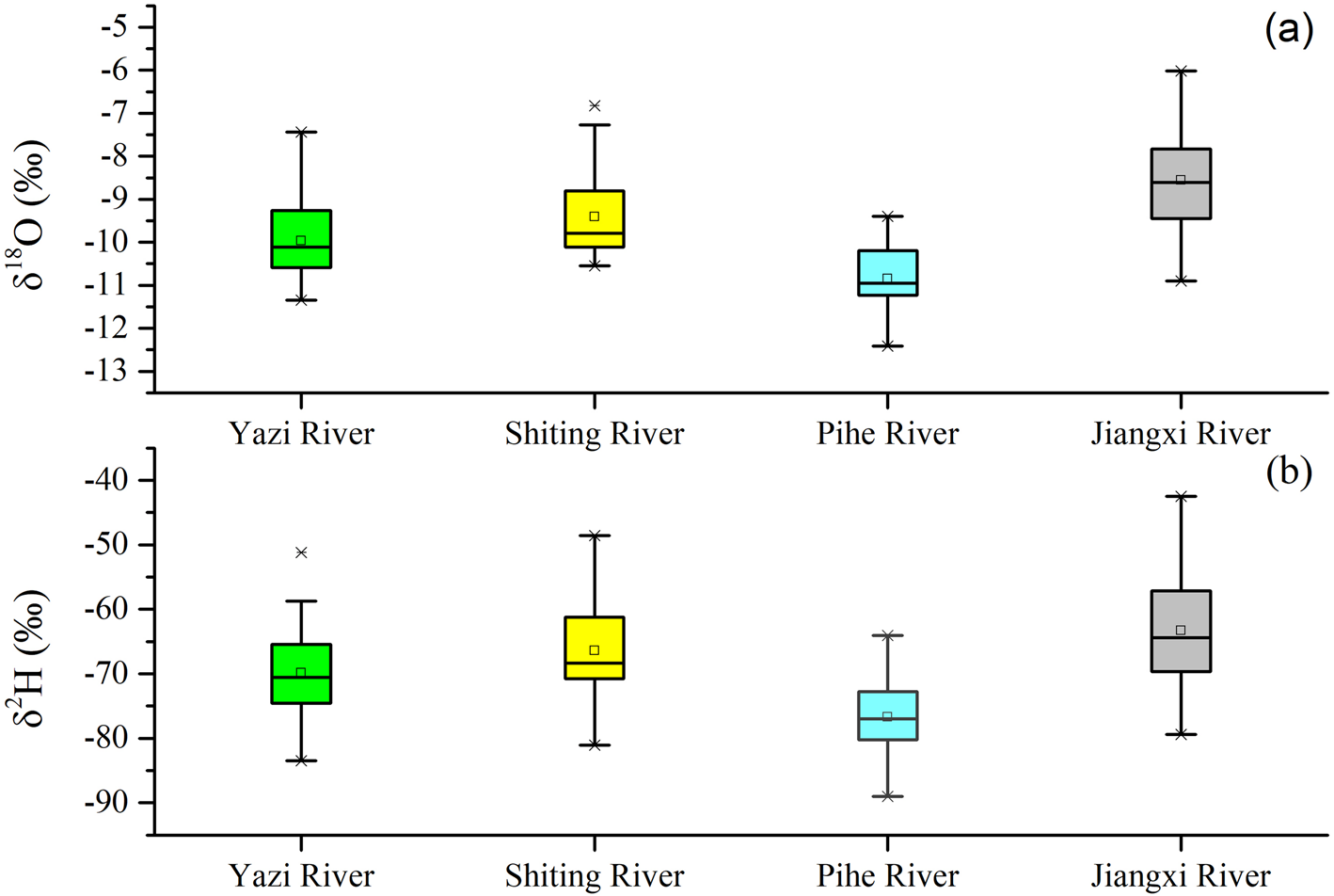
Box plots of spatial variation for δ^18^O (**a**) and δ^2^H (**b**) among Yazi River, Shiting River Pihe River and Jiangxi River. The box plot has lines at the lower quartile, median, and upper quartile values. The whiskers of the box are lines extending from each end of the box to show the extent of the rest of the data. The outliers of the box are data with values beyond the ends of the whiskers.

The measured δ^18^O and δ^2^H values of river water collected from outlets of four tributaries (Yazi, Shiting, Pihe and Jiangxi River) range from − 12.4 to − 8.6‰ and − 89 to − 63.3‰, respectively (Fig. 2). The δ^18^O values of Pihe River waters range from − 12.4 to − 9.4‰ and δ^2^H values range from − 89 to − 64‰. The lowest average δ^18^O and δ^2^H values and the lowest standard deviation relative to the other three rivers are observed in the Pihe River water. The average δ^18^O and δ^2^H values of Yazi River are − 9.96‰ and − 69.8‰ respectively which are close to that of Shiting River. The average δ^18^O and δ^2^H values of these two rivers are in general higher than that of the Pihe River and lower than that of Jiangxi River, with a medium standard deviation. The river water from the Yazi River and Shiting River contains a relatively medium magnitude of variations in δ^18^O and δ^2^H, with δ^18^O ranging from − 11.3 to − 7.4‰ and − 10.6 to − 6.8‰ and δ^2^H from − 83.4 to − 51.2‰ and − 81 to − 48.6‰ respectively. The highest δ^18^O and δ^2^H values are observed in the Jiangxi River, with δ^18^O ranging from − 10.9 to − 6.0‰ and δ^2^H from − 79.4 to 42.5‰. The standard deviation of δ^18^O and δ^2^H values in Jiangxi River is high as it reaches 1.14‰ and 8.97‰ respectively.

The lowest isotopic composition of the Pihe River shows a different water source between Pihe River and the other three tributaries. Pihe River receives the water from the Min River where the isotopic composition of surface water proved depleted^37^. The river water is generally a mixture of channel precipitation, overland flow, subsurface baseflow, agricultural drains and even municipal effluents and its isotope values could be viewed as the average level of the comprehensive characteristics of the catchment as well as the reflection of the climate condition. The close isotope values of the Yazi River and Shiting River indicate the similar characteristics of the catchment and climate condition. Except for the Pihe River, another three Rivers’ isotope value increase with the latitude and elevation, which could be attributed to the precipitation’s latitude effect that causes the depleted isotope values in precipitation. Jiangxi River is a seasonal river heavily affected by precipitation. Hence, the δ^18^O and δ^2^H values of Jiangxi River water are also affected by the high δ^18^O and δ^2^H values of precipitation. Moreover, the construction of reservoirs in Jiangxi River increases its water evaporation phenomena and results in its enriched isotope values^38^.

### Characteristics of the river water line (RWL) among four sub-catchments

**Figure 3:**
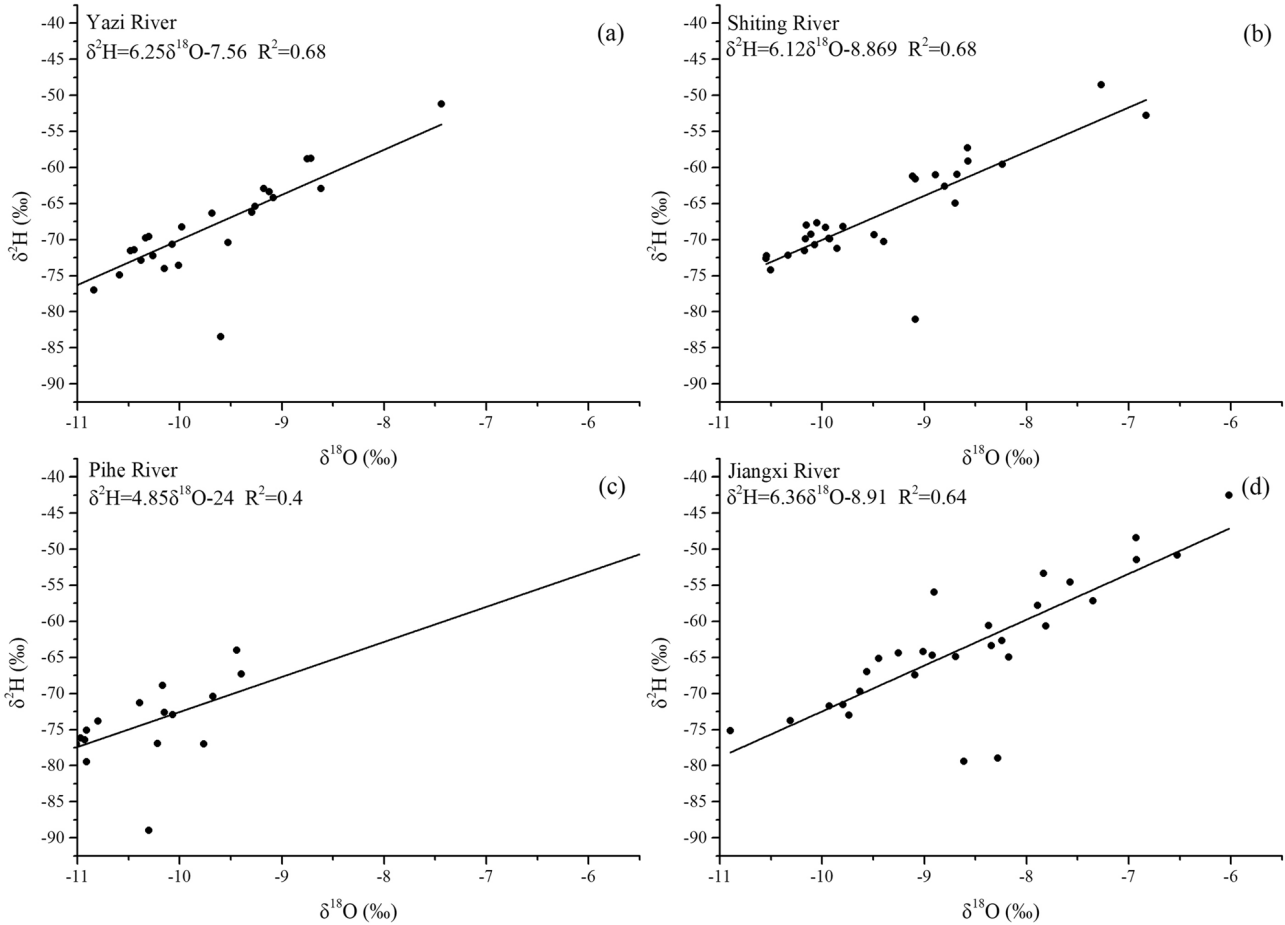
RWL of four tributaries of the Tuojiang River (Yazi River (**a**), Shiting River (**b**), Pihe River (**c**), Jiangxi River (**d**)).

Based on the stable isotope data measured on water samples collected from four tributaries, we calculate the δ^18^O versus δ^2^H relationship (i.e. river water line) of the four rivers (Fig. 3). The slopes of the RWL for the Yazi River, Shiting River, Pihe River and Jiangxi River range from 4.85 to 6.36. Compared to the slope of the local meteoric water line (LMWL) of 7.61 in the study area, the slope and intercept of the RWL of four tributaries are low^39^. What’s more, compared with the slope of RWL in adjoining areas, such as the Hailuogou River and the Heishui River in Sichuan Basin, the slopes of RWL in the four sub-catchments are still low^37,40^. The lower slopes of the four RWL indicate that the precipitation occurs evaporation before reaching the river. The stable isotope in the precipitation undergoes kinetic fractionation which makes different fractionation factors between δ^2^H and δ^18^O, then eventually leads to the decrease of the slope of RWL. The lowest slope of RWL is observed in the Pihe River and it could be attributed to the high evaporation of the Pihe River water. The evaporation ratio of the Pihe River water, according to previous studies, ranging from 0.48 to 10.22%. There are seasonal differences in evaporation, water evaporates significantly in the rainy season under the influence of high temperature^27^. The slopes of RWL of the other three rivers are much larger than those of the Pihe River. Spatial heterogeneity of physio-geographical components leads to spatial and temporal changes of the hydrological process in the catchment. The different slopes of RWL between four tributaries of the Tuojiang River reflect the characteristics of each catchment. The largest area and lowest slope of Pihe River catchment may slow down the rate that precipitation flows into the river, resulting in the long residence time and greater evaporation of the water in the Pihe River catchment. Thus, make the slope of the RWL of the Pihe River smaller than the other three rivers. The difference of isotope value and slope of the RWL among four catchments reflect different hydrologic features of four sub-catchments. It also proves that the stable isotope could be used as a tracer to investigate the characteristics of the catchment and enhance our understanding of the hydrological process in the catchment.

### Seasonal analysis of δ^18^O patterns and estimates of MRT among four sub-catchments

**Figure 4:**
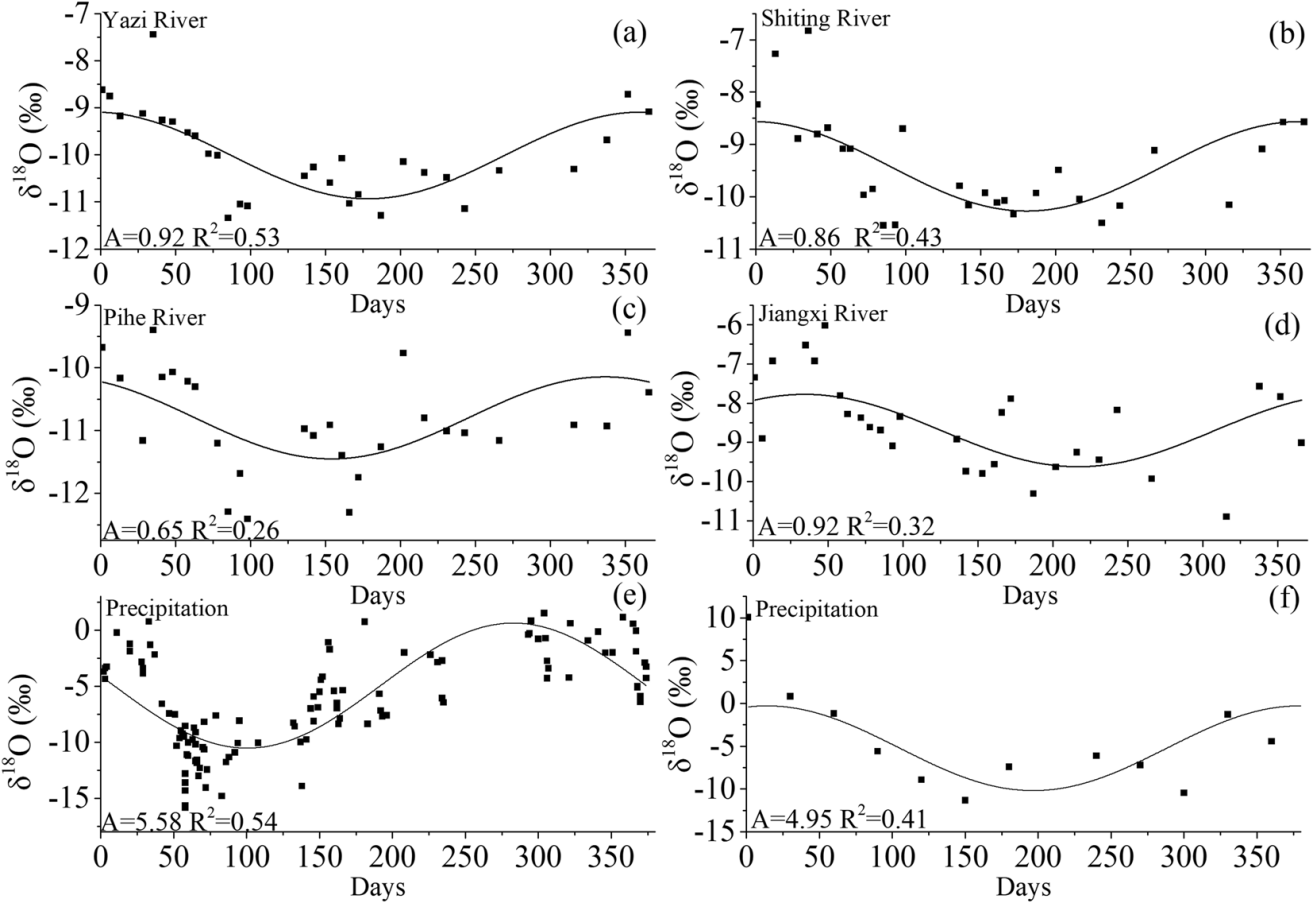
Fitted annual regression models to δ^18^O for precipitation (**e**) and river water (**a**–**d**) during 2018–2019. (**f**) is the fitted annual regression models to δ^18^O for precipitation at Chengdu City during 1996–1997.

The four sub-catchments are located in southwest China where belongs to the subtropical monsoon climate zone. It is controlled by the East Asian monsoon, the Indian monsoon, and the northwest monsoon. Therefore, significant seasonal δ^18^O trends in precipitation and river water are observed here (Fig. 4). Previous studies prove that the sine-wave approach could be used to estimate MRT by using a lumped parameter model that takes advantage of the strong seasonal changes in the isotopic composition of precipitation^41,42^. The seasonal δ^18^O trends of precipitation and river water could be interpreted quantitatively by using periodic regression analyses (Eq. (4)) (Fig. 4). Although the modelled curves simplify the pattern of isotopic changes in precipitation and river water, the results are all nonetheless statistically robust (p < 0.02). The correlation between measured and modelled δ^18^O in precipitation and river water is relatively strong, with r^2^ = 0.54, 0.53, 0.43, 0.26, 0.32 for precipitation, Yazi River, Shiting River, Pihe River and Jiangxi River respectively. These results are comparable with other similar studies^18,43^. The lowest amplitude and relatively weak r^2^ of 0.26 are observed in the Pihe River and it could be attributed to the existence of Pihe Water Supply Project. The incoming water of the Pihe Water Supply Project is dispatched by the Zipingpu Reservoir to meet the water consumption of the Dujiangyan Irrigation District. Then the remaining water is supplied to the Pihe River catchment. During this process, the seasonal change of the isotope value of river water is disturbed by human activities. Modelled δ^18^O values for the Yazi River, Shiting River, Jiangxi River fit better with measured values and show larger δ^18^O amplitude values, ranging from 0.86 to 0.92 (Table 3). Combined with a large amplitude, these significant seasonal variations in δ^18^O of the Yazi River, Shiting River and Jiangxi River reflect greater responsiveness to recent precipitation inputs. The δ^18^O amplitude of precipitation (A = 5.58) during 2018–2019 is similar to the δ^18^O amplitude of precipitation during 1996–1997 in Chengdu. The amplitude of the sine curves can be used to estimate catchment MRT by Eq. (5). Shorter MRTs (346 to 374 days) are observed in the Yazi River catchment, Shiting River catchment and Jiangxi River catchment While longer MRT (493 days) is observed in the Pihe River catchment. According to Fenicia et al.^44^, the traditional convolution-based mixing model based on steady-state flow assumption affect the estimation of MRT for high temporal resolution simulations (e.g. hourly). But, this assumption still provides insight for estimating MRT by using long-term averaged data (e.g. monthly)^44^. For example, Ogrinc^18^ estimated the MRT of the Sava River catchment by using monthly isotope data. The result shows a longer MRT (2.2 years) than our study region. The seasonal variation of stable isotope oxygen-18 is also used to estimate MRT in central Pennsylvania. MRTs obtained for river water were 9.5 and 4.8 months which is short compare to the result of our study^45^.

**Table 3:** Residence times (days) estimated from regression analysis for isotope data of precipitation and river water.

Name	MRT (days)	Amplitude (%)	N	R^2^	RMSE	P-value
Yazi	348	0.92	30	0.53	0.61	0
Shiting	375	0.86	29	0.43	0.67	0
Pihe	493	0.65	28	0.26	0.67	0
Jiangxi	346	0.92	29	0.32	0.91	0
Precipitation		5.58	113	0.54	3.06	0

N is the number of water samples. RMSE is the root mean square error of regression.

### Relationship between topography, soil and MRT

**Figure 5:**
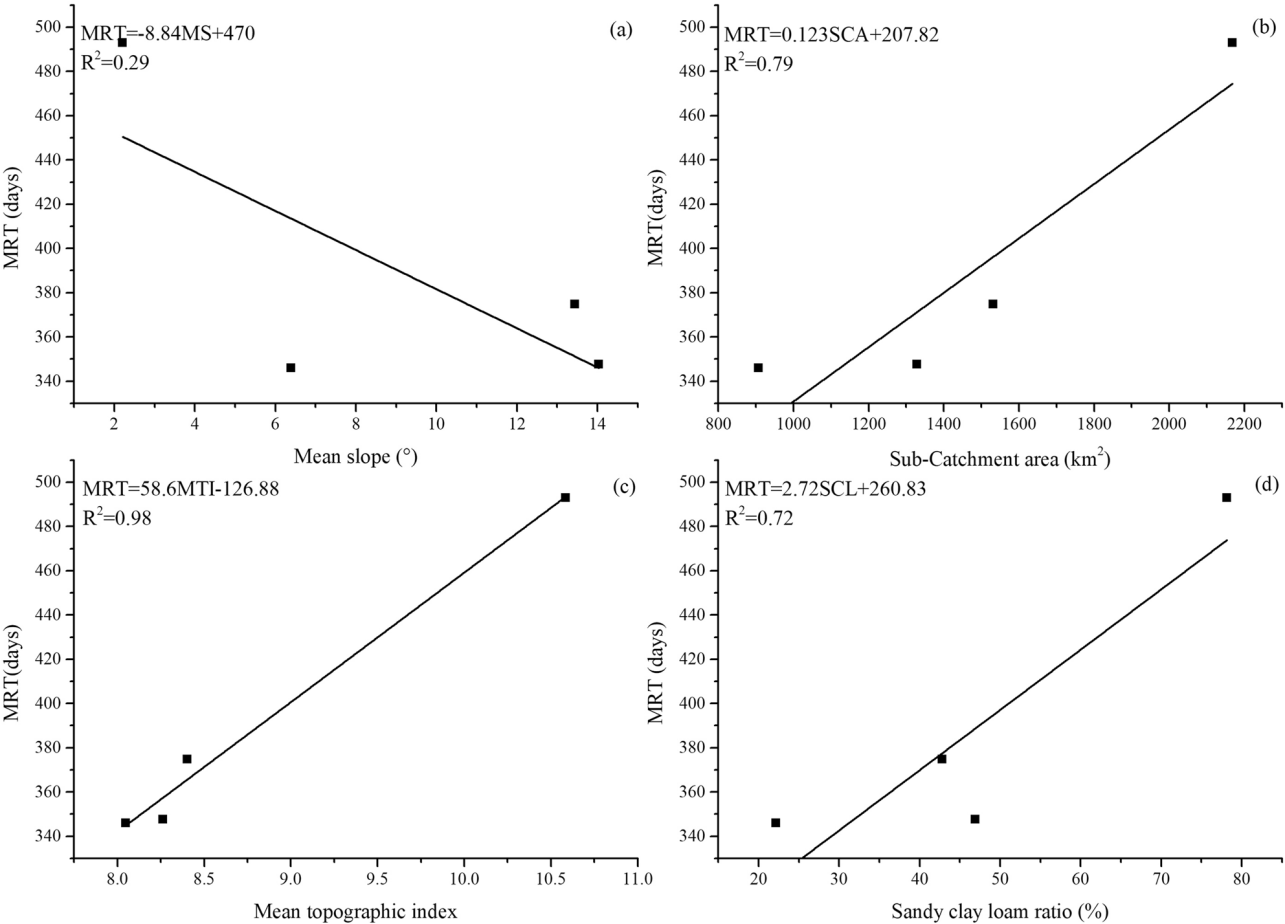
Relationships between mean slope (**a**), sub-catchment area (**b**), mean topographic index (**c**), sandy clay loam ratio (**d**) and MRT.

A 90 m digital elevation model (DEM) is used to compute topographic attributes for 4 sub-catchments in the Tuojiang River catchment. The relationships between topography, soil and estimated residence time are examined in a formal manner by correlation analysis (Fig. 5). Regression equations for mean slope (MS), sub-catchments area (SCA), mean topographic index (MTI) and sandy clay loam ratio (SCL) are shown in Eqs. (6), (7), (8) and (9), respectively.

6 $$\text{MRT}=-8.84\text{MS}+470({\text{R}}^{2}=0.29)$$

7 $$\text{MRT}=0.123\text{SCA}+207.82({\text{R}}^{2}=0.79)$$

8 $$\text{MRT}=58.6 \text{MTI} -126.88 ({\text{R}}^{2}=0.98)$$

9 $$\text{MRT}=2.72 \text{SCL}+260.83 ({\text{R}}^{2}=0.72)$$

The relatively weak negative correlation between the mean slope of sub-catchments and MRT is observed in this region (r^2^ = 0.29). The negative relationship between slope and MRT is consistence with our expectation with the increase of gravitational potential. This negative correlation has also been observed in other regions, for example, in Zapotlán catchments (r^2^ = 0.91)^17^. A relatively weak correlation between slope and MRT could be attributed to the gentle terrain in the study region. In some studies, the positive relationships between slope and MRT are also found, for example, in Feugh catchment (r^2^ = 0.67) and Feshie catchment (r^2^ = 0.48)^41,43^. This positive relationship between slope and MRT in the Feugh catchment and Feshie catchment may be influenced by the most responsive peat soils on the flatter hilltops and the steeper slopes. So, the weak correlation between slope and MRT in our study area may also be disturbed by another factor. In our study, the sub-catchments area shows an apparently positive relationship to the estimated MRT (r^2^ = 0.79). This result is consistent with some previous studies^32,46^. However, a weak relationship between catchment area and MRT is also observed in other studies^7,8^. These two different results are likely because of other features on MRT of the catchment that offset the effect of the area. Most of the strong relationships between catchment area and MRT are observed in a catchment with gentle slope^32^ while most weak relationships between catchment area and MRT are observed in a catchment with steep slope^8,43^. This preliminary finding indicates that the area of the catchments may play a major role in controlling MRT in the plain with gentle terrain. While in the mountainous region with steep terrain, the slope of the catchment may play a major role in controlling MRT.

In order to find more effective topographic factors that affect MRT, another topographic attribute, topographic index (i.e., $\ln(a/{\tan}{\beta})$), is computed by the geographic information system (GIS). Where *a* is upslope accumulated area and $\beta$ is the local slope angle^47,48^. The strongest positive relationship between topographic index and MRT is observed here (r^2^ = 0.98). The similar strong correlations between MRT and topographic index have also been found in other studies^17^. The mean value of topographic index does not vary significantly among Yazi, Shitingjiang and Jiangxi River catchments (from 8 to 8.4). The highest value of topographic index, observed in Pihe River catchment, is 10.6 (Table 1). The topographic index $\ln(a/\tan{\beta})$ is an important component of many physically based geomorphic and hydrologic models as it reflects the spatial distribution of soil moisture, surface saturation and runoff generation processes^47^. For catchment in humid areas like our study region, the runoff is dominated by stored-full runoff, and the runoff is mainly generated in regions where the soil moisture reaches above the field capacity—contributing areas. Previous studies^19,49,50^ suggest that MRT is positively correlate with the contributing area. With the increase of contributing areas and the decrease in slopes, the topographic index will increase too. So, as the topographic index increases, its MRT will also increase.

Soil is an important part of catchment hydrology and its physical properties like moisture retention, distribution of pore space and specific water capacity have an important influence on the catchment’s response to precipitation. These physical properties are related to the texture of the soil. In some regions like glaciated mountains, soil hydrology can also be a useful predictor of MRT due to the fact that its complex drift distributions may over-ride topographic and geological differences^1^. Therefore, the sand clay loam ratio is calculated in the four sub-catchments to investigate the possible relationship to MRT. The results show that the proportion of sandy clay loam has a relatively strong positive relationship with MRT (r^2^ = 0.72). Sandy clay loam in the study region has a certain number of large pores and a considerable number of capillary pores. So, it has good ventilation, water permeability and good water retention performance and it is mostly used for agricultural cultivation. Its good water permeability increases the vertical movement of water and the flow path length, resulting in long MRT. Also, its good water holding capacity slows the catchment response to the precipitation, which increases its MRT. The longer the retained water in the soil, the stronger its evaporation, which results in the enrichment of its isotope value in water^51^. This phenomenon is also found in river water in the study region and the slope of its RWL gradually decreases with the increase of MRT. The influence of topographic factors on MRT has been confirmed in many articles^36,41^. Most of them believe that some descriptions of topography provide a first-order control on flow processes and transport. The influence of topography on MRT has also been verified in this study. However, in the plain region where the topographical relief is not significant, the catchment area has a greater impact on the MRT than the catchment slope. Therefore, when we establish the MRT model, we should pay attention to the influence of the area factor on MRT in the plain region. In addition, our research shows that the topographic index is very closely related to MRT, which indicates that the topographic index is a reliable indicator for MRT simulation.

### Preliminarily exploration of the relationship between land use type and residence time

**Figure 6:**
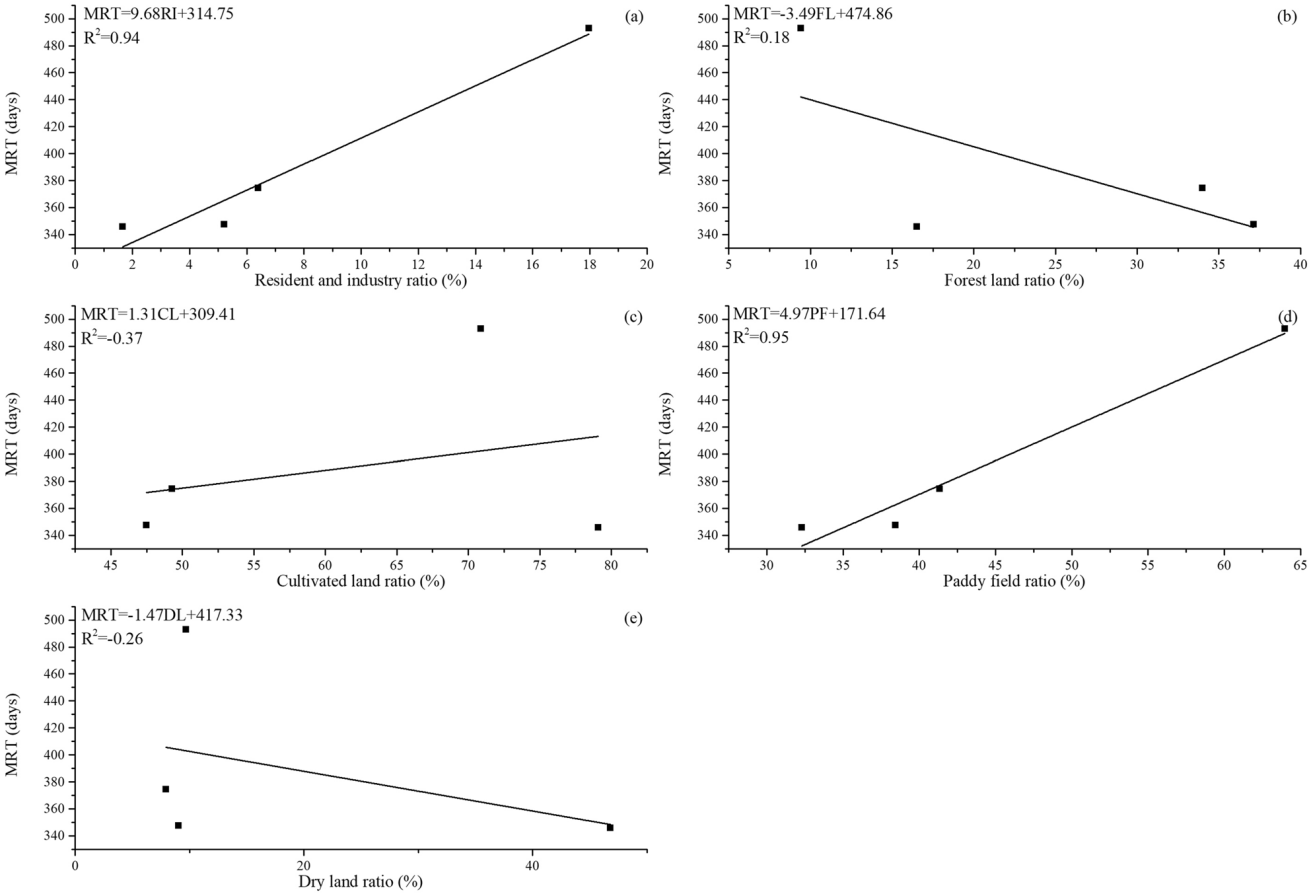
Relationships between resident and industrial area ratio (**a**), forest land ratio (**b**), cultivated land ratio (**c**), paddy field ratio (**d**), dry land ratio (**e**) and MRT.

Land use types are closely related to groundwater and runoff processes in the catchment and are susceptible to human activities^52^. Additionally, the residence time can be used as a proxy to understand the hydrologic sensitivity to land use^7^. As discussed above, the catchment area has a relatively strong relationship with MRT. Here, the catchment area is classified according to land use type to explore Which land types impose a greater impact on MRT. In the four sub-catchments of the study region, the river network is densely covered and the terrain is relatively flat. The four tributaries of the Tuojiang River pass through many towns and agricultural irrigation regions, which are affected greatly by human activities. The land use types in the study region include forest land, resident and industrial area, water area, grassland, and cultivated land. The cultivated land includes paddy fields and dry land. In this study, the ratios of resident and industrial area (RI), forest land (FL), cultivated land (CL), paddy field (PF) and dry land (DL) versus total sub-catchment area are calculated to explore the potential relationship between land use type and MRT (Fig. 6). The results are divided into three categories including strong correlation, weak correlation and non-correlation. Strong positive correlations are observed between paddy field ratio, resident and industrial area ratio and MRT (r^2^ > 0.9). A positive relationship is observed between MRT and resident and industrial area ratio though it is counter-intuitive; increased resident and industrial area ratio would lead to decreasing MRT. Increases in the resident and industrial area ratio leads to a large proportion of impervious areas and the infiltration from the ground surface is close to zero. Stored-full runoff and slope runoff occur relatively quickly. Precipitation fills every depression and surface runoff formed quickly, resulting in a fast response to the rainfall in relevant catchments^53^. However, high resident and industrial area ratios of four sub-catchments generally have a more paddy field ratio. Moreover, resident and industrial area ratios are very small in four sub-catchments, which has a limited impact on the hydrological condition of the entire catchment (Table 2). Therefore, the impact of the paddy field ratio on MRT may mask the impact of the resident and industrial area ratio on MRT. As a result, the relationship between MRT and the resident and industrial area ratio shows a trend that contradicts our expectation. Four sub-catchments are described as major grain-producing regions in Sichuan Province. Paddy field accounts for a large proportion, ranging from 32.3 to 64% in four sub-catchments. The impact of paddy fields on the MRT mainly includes two parts, one is the interception and evaporation of precipitation by planting plants, and another one is the soil affecting the infiltration and confluence of precipitation. Paddy fields in the study region are mainly planted with rice that its transpiration and interception evaporation is significant. The paddy soil in the study area is mainly sandy clay loam, which has good water retention performance. Therefore, as the proportion of paddy fields in the catchment increases, its MRT grows higher. A weak negative correlation is observed between forest land ratio and MRT (r^2^ = 0.18) (Fig. 6). This may be attributed to the low vegetation coverage of the Tuojiang River catchment and its soil properties in forest land. For example, among the four sub-catchments, the forest coverage of the Shiting River catchment and Yazi River catchment is relatively large, ranging from 33.99 to 37.13% (Table 2). Its soil porosity in forest land is large and has strong permeability, but its water retention is poor, which causes the shorter MRT. Non-correlations are observed between cultivated land ratio, dry land ratio and MRT (r^2^ < 0). The results do not conform to our expectation as the study area is a plain region with advanced agriculture and a large ratio of cultivated land area. These results show that in regions dominated by irrigated agriculture, MRT is closely relate to the ratio of paddy field area compared to the total cultivated land area ratio or dry land area ratio. These results also illustrate that in future research on the MRT model, we should pay attention to the influence of the paddy field on the MRT in catchments dominated by agricultural irrigation. MRT plays an important role in controlling the quality of water that drains soil or catchments^45^. With the advancement of urbanization, many paddy fields have been changed to urban land, which will undoubtedly cause shorter MRT, thereby leads to serious pollution in the catchment. As the hydrological investigation on the catchment scale is very difficult, we only conducted a long-term isotope monitoring on four sub-catchments. The scarcity of sampling sites might result in accidental errors. For example, the r^2^ for cultivated land ratio and dry land are − 0.37 and − 0.26 respectively, which is impossible. It suggested that a small catchment with more sub-catchments in the future research should be selected. More sub-catchments would allow the study to obtain more samples to increase the representativeness of the study. A small catchment is conducive to long-term sampling and reduce the impact of spatial heterogeneity on the MRT. Nevertheless, as an initial attempt to simulate MRT in catchments, this paper provides profound insights and experience for the study in this field.

## Conclusion

In this paper, characteristics of RWL and variations of stable isotope in river water among four sub-catchments in Tuojiang River catchment are investigated. The result shows that Pihe River has depleted isotope value and the lowest slope of RWL. It also indicated different sources between the Pihe River and the other three rivers as well as high evaporation of the Pihe River, which is generally consistent with the long mean residence time. The sine-wave approach is used to model the season trends in δ^18^O and the amplitude of seasonal δ^18^O curves in river water is used to estimate the MRT. Modelled δ^18^O value compares well to measured δ^18^O value in river water from different locations within the Tuojiang River catchments. Estimated MRT within four sub-catchments ranges from 346 to 493 days and the longest residence time is observed in Pihe River catchment. Moreover, potential relationships between topography, catchment area, soil, land use and MRT are investigated in this paper. The results show that the topographic feature is a main affecting factor of MRT, while the influence of topographic factors on the correlation between slope and MRT in the plain region is relatively weak. A relatively strong positive relationship between catchment area and MRT is observed in the study region which suggests that the catchment area may have a greater impact on the MRT than the slope in plain region. We should pay more attention to this in the future MRT simulation of the catchment in the plain region. In addition, the soil type is also an important parameter in simulating catchment MRT. Compared with other factors, the topographic index may be a reliable parameter to estimate the MRT of the catchment as its strongest correlation between topographic index and MRT. By comparing the correlation between land use types and MRT, we find that in plain region with developed agricultural irrigation, the proportion of paddy fields in the catchment has a great impact on MRT. Results from this study could reveal several catchment characteristic parameters related to MRT, which will provide some reference for future MRT models and improve our understanding of hydrological and hydro-chemical processes in the catchment (Supplementary Information).

## Supplementary Information

Supplementary Information.

## Data Availability

The dataset is provided by National Cryosphere Desert Data Center (http://www.ncdc.ac.cn).
