# Supplementary material for: Using stable isotopes as tracer to investigate hydrological condition and estimate water residence time in a plain region, Chengdu, China
Source: Sci Rep. 2021 Feb 2;11:2812. doi: 10.1038/s41598-021-82349-3 (PMC7854618; doi:10.1038/s41598-021-82349-3)
Supplement: Supplementary file 1 — Supplementary Information. [file 41598_2021_82349_MOESM1_ESM.docx]

**Using stable isotopes as tracer to investigate hydrological condition and estimate water residence time in a plain region, Chengdu, China**

Jing Zhou^a,b^, Yuchuan Meng^a,b*^, Guodong Liu^a,b^, ChengCheng Xia^a,b^, Ke Chen^a,b^, Yu Chen^a,b^

^a^State Key Laboratory of Hydraulics and Mountain River Engineering, Sichuan University, Chengdu 610065, China

^b^College of Water Resources and Hydropower, Sichuan University, Chengdu 610065, China

*Corresponding author: 545001616@qq.com

|  | Precipitation | | River water | |
| --- | --- | --- | --- | --- |
| Month | δ^18^O (‰) | δ^2^H (‰) | δ^18^O (‰) | δ^2^H (‰) |
| 5 | -2.7299 | -9.3604 | -8.6906 | -60.105 |
| 6 | -8.0278 | -54.929 | -8.6928 | -61.879 |
| 7 | -12.105 | -85.95 | -9.8346 | -73.752 |
| 8 | -10.964 | -80.291 | -10.81 | -71.104 |
| 9 | -10.2 | -67.539 | -10.195 | -71.235 |
| 10 | -6.6988 | -40.867 | -10.389 | -73.034 |
| 11 | -7.0567 | -48.938 | -10.585 | -74.679 |
| 12 | -4.6378 | -19.833 | -10.116 | -71.068 |
| 1 | -4.662073 | -15.017294 | -9.9519 | -70.208 |
| 3 | -3.0863 | -6.474 | -9.8772 | -68.083 |
| 4 | -0.8308 | 0.99658 | -9.0959 | -62.346 |
